# Stable coherent mode-locking based on $\pi$ pulse formation in single-section lasers

**DOI:** 10.1038/s41598-020-80775-3

**Published:** 2021-01-13

**Authors:** Rostislav Arkhipov, Anton Pakhomov, Mikhail Arkhipov, Ihar Babushkin, Nikolay Rosanov

**Affiliations:** 1 St. Petersburg State University, Universitetskaya nab. 7/9, St. Petersburg, 199034 Russia; 2 ITMO University, Kronverkskiy Prospekt 49, St. Petersburg, 197101 Russia; 3 Ioffe Institute, Politekhnicheskaya str. 26, St. Petersburg, 194021 Russia; 4 Institute of Quantum Optics, Leibniz University Hannover, Welfengarten 1, 30167 Hannover, Germany; 5 Max Born Institute, Max-Born-Strasse 2a, 10117 Berlin, Germany; 6 Cluster of Excellence PhoenixD (Photonics, Optics, and Engineering-Innovation Across Disciplines), Welfengarten 1, 30167 Hannover, Germany

**Keywords:** Mode-locked lasers, Ultrafast lasers, Ultrafast photonics

## Abstract

Here we consider coherent mode-locking (CML) regimes in single-section cavity lasers, taking place for pulse durations less than atomic population and phase relaxation times, which arise due to coherent Rabi oscillations of the atomic inversion. Typically, CML is introduced for lasers with two sections, the gain and absorber ones. Here we show that, for certain combination of the cavity length and relaxation parameters, a very stable CML in a laser, containing only gain section, may arise. The mode-locking is unconditionally self-starting and appears due to balance of intra-pulse de-excitation and slow interpulse-scale pump-induced relaxation processes. We also discuss the scaling of the system to shorter pulse durations, showing a possibility of mode-locking for few-cycle pulses.

## Introduction

Mode-locking is a method to obtain short pulses directly from laser oscillators^1–4^. It is a common and very basic technique, used in virtually all areas of modern optics. Typical for applications is so called passive mode-locking (PML), achieved by incorporating a nonlinear (saturable) absorber with suitable properties into the laser cavity. In such two-section cavities, generation of short pulses is achieved due to saturation of the amplifier/absorber section, and thus the pulse duration $\tau _p$ is larger than the polarization relaxation time $T_2$ in the amplifier and absorber sections. Hence, in such PML-based lasers, pulse duration is fundamentally limited by the inverse bandwidth of the gain medium^1,2,5^. Opposite situation arises, when the electric field in the cavity is so strong, that the Rabi frequency *Ω*_R_^6^ is larger than the inverse dephasing time of the medium, $\Omega _{R} \gg \frac{1}{T_2}$. In this case, the pulse duration is typically smaller than the dephasing time, $\tau _p < T_2$, and the light-matter interaction taking place on the time of the pulse duration is thus “coherent”, so the mode-locking appeared there is often called “coherent mode-locking” (CML). The basic features and key differences between standard PML and CML are summarized in the Table 1, where by *T*_1_ the population relaxation time is denoted.

**Table 1:** Standard (incoherent) passive mode-locking (PML) and coherent mode-locking (CML).

Standard passive mode-locking (PML)	Coherent mode-locking (CML)
Based on incoherent ($\tau _p> T_2$) gain/absorption saturation	Based on Rabi oscillations
$\Omega _R<1/T_2$, $T_2<\tau _p<T_1$	$\Omega _R>1/T_2$, $\tau _p<T_2<T_1$
Significant part of energy is left in absorber	Almost no losses in absorber ($2\pi$-pulse of SIT)
Only part of energy is taken from the amplifier	Almost all energy stored in the amplifier is taken ($\pi$ pulse)
$\tau _p$ is fundamentally limited by $T_2$	$\tau _p$ can be much smaller than $T_2$
Few-cycle pulses are possible only in a broadband media	Few-cycle pulsese are possible in narrowband media
$\tau _p$ typically increases with increase of the pump power	$\tau _p$ decreases with the pump power

The key idea of CML^5,7,8^ is to use the gain and absorber sections both in the coherent regime ($\tau _p < T_2$). In the absorber section, a pulse of self-induced transparency (SIT)^6,9,10^ (a $2\pi$ pulse) is formed. Such a pulse is a solitary wave, which stably propagates in the absorber without losses. As such, it is also stabilized against perturbations, in particular against instabilities of the non-lasing state. The gain section, in contrast, has to be arranged in such a way, that essentially the same pulse has an area $\pi$ instead of $2\pi$. Besides, it is assumed that the gain section is nearly fully inverted at the moment when the pulse arrives. The $\pi$ pulse is a “half of Rabi oscillation” and thus it returns all the atoms of the gain section to the ground state, so that the energy is fully transferred from the medium to the pulse. The resulting pulse duration was predicted to be able to achieve even the single-cycle level^11,12^. This is in agreement with the theoretical prediction^11–13^ and experimental demonstration^14^ of Rabi-oscillations and other SIT-based pulses at few- and single-cycle level. CML potentially allows passive mode-locking in quantum cascade lasers^15–17^, which is known to be otherwise virtually impossible because of too fast carrier relaxation times^18^. Besides, CML can arise if the absorber section works in the coherent regime^19–21^ whereas the amplifier section is in the saturable regime. This type of CML was recently demonstrated experimentally^22–25^. In^26^, it was shown that CML should arise as a very stable and even self-starting regime if the cavity round-trip time is of the order of $T_1$, allowing the medium to relax enough in between the pulses.

One of the signatures of CML making them different from PML, is that the pulse duration decreases with increasing the output power^24,25^. In the case of the coherent absorber section, this is also easy to understand from the dynamics of the gain medium alone. Namely, as it is known, the pulse propagating in an amplifier (in a propagation, not a cavity geometry) shortens its duration, whereas the pulse amplitude increases while maintaining the constant $\pi$ area^6,10,27^. That is, returning to the cavity, more energy is available to the pulse in the gain section, shorter its duration becomes in the CML regime. This property takes place for the CML with the only absorber section in the incoherent regime. The latter was recently experimentally demonstrated in^24,25^.

On the other hand, from the very early days of laser physics it has been known, that a single-section laser can demonstrate self-pulsations and mode-locking^28^, that is, the absorber section is not absolutely necessary. It was also realized^29–31^, that self-pulsations in single-section lasers should appear via an universal bifurcation scenario, where the CW regime, arising at the lasing threshold is destabilized at certain pump level often called the “second threshold” (the lasing threshold is then known by “first threshold”). The instability at the second threshold is refereed to as Risken-Nummendal-Graham-Haken (RNGH) one, and develops into pulsations when the intermode interval becomes comparable to the Rabi frequency beatings (thus suggesting that RNGH instability is based on coherent interaction).

The RNGH instability threshold takes place at very high pump levels and, at the same time, long cavities. In most realistic situations, especially in solid state lasers, it can never be reached; instead, the CW regime is destabilized much earlier due to various further reasons, leading also to self-pulsations, mode-locking, or to more complicated regimes. In particular, mode-locking and self pulsations in single-section lasers have been observed in bulk semiconductor^32–36^, quantum well^37–40^, quantum cascade^41,42^, quantum dash^43,44^, and quantum dot^45–48^ lasers. The mechanisms responsible for the mode-locked operation in these lasers were identified as four-wave mixing^40^ or spatial hole burning^49^ (see also^50^ and references therein). These mechanisms, in contrast to RNGH instability, do not assume coherent interaction, that is, no nontrivial atomic polarization dynamics develops. This make them different from the CML, where the dynamics of atomic polarization plays the decisive role.

However, self-pulsations in single-section gas lasers, accompanied by coherent effects, were observed experimentally by several authors^51^, since gas lasers allow long cavities, large gain, and huge decoherence times $T_2$. In particular, Fox and Smith^51^ observed pulsations in He-Ne laser and proposed that they were related to $\pi$ pulse formation. Experimentally observed values of pulse durations were in agreement with theoretical estimations for $\pi$ pulses and reduction of pulse duration with increase of lasing power was observed as further indication of coherent dynamics^6^. Furthermore, Harvey et al.^52^ observed $\pi$ pulses and mode-locking in argon-ion laser. Inverse proportionality of the pulse duration to the power was observed in this experiment as well. Dudey et al.^53^ investigated coherent effects in argon-ion laser operated in a high-Q-cavity configuration. They recognized some features of coherent pulse propagation such as coherent ringing. Casperson and co-workers^54,55^ reported both experimental and theoretical analysis of self mode-locking in xenon laser and observed similar coherent effects and harmonic mode-locking regimes with the increase of cavity length. Theoretical modeling performed in^56^ corroborated experimental studies performed earlier in Xe-laser. Even in solid state (semiconductor) optical amplifier, coherent effects were observed recently in^57–59^.

As it was mentioned above, the absorber section in two-section CML lasers plays the fundamental role since it provides stabilization of the mode-locked pulses via formation of a SIT-soliton. In this paper we show that, surprisingly, under certain conditions, a very stable, self-starting CML is possible without such absorber-based stabilization, that is, in a single-section travelling-wave laser, giving pulses with the area close to $\pi$ in the gain section. Appearance of stable self-starting CML is possible for proper combination of the cavity length and relaxation constants, and is rooted in the self-tuning of the pulse duration and energy in such a way, that intra-pulse de-exitation dynamics is matched to inter-pulse pump-induced excitation. We show, that the mode-locking develops via RNGH instability, at rather high pump excesses above threshold. In our case the CW regime remains stable before RNGH, as well as the mode-locking regime after it. To analyze the possibility and features of CML we use a diagram technique based on the McCall and Hahn area theorem^5,6,9,60^ to study inhomogeneously broadened media. To study homogeneously broadened media we use numerical simulations of the Maxwell-Bloch (MB) laser equations. We compare the dynamics of single-section laser with two-section laser containing coherent absorbing medium and show that in certain parameter region single-section laser has similar lasing parameters than the two section laser, so introducing the second section for the mode-locking stabilization is not necessary. Finally, we find a rescaling of the laser parameters, leaving the dynamics invariant as the pulse duration changes, and using it, establish conditions for generation of few-cycle pulses.

The article is organized as follows: in “Coherent pulse propagation and area theorem” section we introduce the area-theorem-based diagram technique; in “CML in a single-section laser and the area theorem” section we use it to show the possibility of CML in inhomogeneously broadened media; in “Numerical simulations” we study homogeneously broadened media via direct numerical solution of the MB equations; in “Condition for few-cycle pulse generation in single-section laser” section we derive the conditions of few-cycle pulse generation; finally, in “Conclusions” section we discuss the results and draw the conclusions.

## Coherent pulse propagation and area theorem

An important quantity describing the pulse dynamics in the coherent regime is the pulse area, defined as^9^

1 $$\begin{aligned} \Phi (z)=\frac{d_{12}}{\hbar } \int _{-\infty }^{+\infty } {\mathscr {E}}(t',z) dt', \end{aligned}$$

where $d_{12}$ is the transition dipole moment of a two-level atom, and ${\mathscr {E}}(t,z)$ is the pulse envelope. Coherent pulse propagation in an amplifying or absorbing inhomogeneously broadened medium is described using so-called area theorem^6,9,10^:

2 $$\begin{aligned} \frac{d\Phi }{dz}=\frac{\alpha _0}{2} \sin \Phi , \end{aligned}$$

with

3 $$\begin{aligned} |\alpha _0| = \frac{8\pi ^2 N_0 d_{12}^2 \omega _0 g(0)}{\hbar c}, \end{aligned}$$

where $\alpha _0$ is the absorption ($\alpha _0<0$) or gain ($\alpha _0>0$) coefficient per unit length, $N_0$ is the concentration of two-level atoms, $\omega _0$ is the medium transition frequency and $g(\Delta \omega )$ is the inhomogeneously broadened spectral distribution function, centered at $\omega _0$, so that $\int _{-\infty }^{+\infty } g(\Delta \omega ) d\Delta \omega$ = 1. Equation (2) is derived assuming the following conditions to hold^6,9,10^:

$$\begin{aligned} T_2^* \ll \tau _p \ll T_2, \end{aligned}$$

where $\tau _p$ is the duration of the generated pulse, $1/T^{*}_{2}$ is the half-width of the inhomogeneously broadened line of the resonant medium and $1/T_2$ is the half-width of the homogeneously broadened line of a two-level atom. That is, it is assumed, that on the pulse duration the individual dipoles belonging to different atomic sub-ensembles, dephase. In particular, Eq. (2) is not valid for a homogeneously broadened media. On the other hand, in the limit of a small signal and thus small area ($\sin \Phi \approx \Phi$), Eq. (2) describes then an exponential decay or growth of the pulse area: $\Phi \sim e^{\alpha _0z/2}$. In the case of a homogeneous medium, linearization of Maxwell-Bloch equations near non-lasing state gives very similar growth/decay rate:

4 $$\begin{aligned} |\alpha _0| = \frac{4\pi N_0 d_{12}^2 \omega _0 T_2}{\hbar c}. \end{aligned}$$

**Figure 1:**
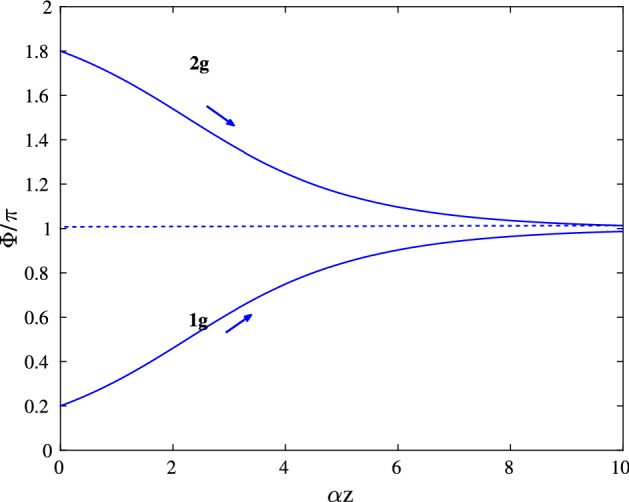
Branches of the solution of Eq. (2) for the amplifier for different initial pulse areas $\Phi _0=0.2\pi$ (curve 1*g*) and $\Phi _0=1.8\pi$ (curve 2*g*) for $|\alpha _0| = 10$ cm$^{-1}$. This figure was created with Matlab R2016b (http://www.mathworks.com).

The solution of Eq. (2) is:

5 $$\begin{aligned} \tan (\Phi /2)=\tan (\Phi _0/2)e^{\alpha _0 z/2}, \end{aligned}$$

where $\Phi _0$ is the initial pulse area. One can see, that, apart from the trivial solution $\Phi = 0$, the area of a stationary SIT soliton is $\Phi = 2 \pi m$ for any positive integer *m*. Two branches of the solution of the area theorem for an amplifying medium are plotted in Fig. 1. In this case, the initial pulse of the area $0< \Phi _0 < 2 \pi$ approaches the steady-state, having the pulse area $\pi$ as the pulse propagates in the medium. At the same time, the pulse duration decreases. In the next section, we will use this approach to study the pulses arising inside a cavity.

## CML in a single-section laser and the area theorem

**Figure 2:**
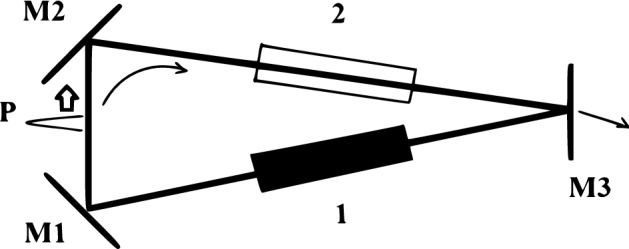
Schematic representation of a mode-locked single-section laser with a ring cavity and unidirectional counterclockwise lasing. 1—amplifying medium, 2—nonreciprocal element, P-pulse travelling in the cavity, M1, M2, M3—cavity mirrors. This figure was created with Paint application for Windows.

Here, using the results of the previous section, we develop a diagram technique similar to the one introduced in^60^ for a two-section laser. We consider a CML in a ring-cavity single-section laser, having an amplifying section 1 inside the cavity, and operating in a unidirectional lasing regime, as shown in Fig. 2. The unidirectonal lasing is supported by nonreciprocal element 2.

The analysis of a traveling wave in a ring cavity laser is simple and physically transparent. On the other hand, for a ring cavity, a counter-propagating wave is not suppressed, in contrast to a two-section cavity, where such waves are ruled out by the nonlinear absorber. In practice, unidirectional generation can be set up by using a nonreciprocal intracavity element.

The branches of solutions of Eq. (2) in the amplifier are shown in Fig. 1. Let us assume, that a short pulse with the infinitesimal area $\Phi _0 \ll 1$ passes through an amplifier 1 (see Fig. 2) in the coherent regime, gets reflected from a mirror $M_3$ with the amplitude reflection coefficient *r*, and then enters the amplifier once again. We assume for simplicity, that other mirrors $M_1$ and $M_2$ do not produce any losses. We also suppose, that the pulse travels long enough in the amplifier, such that the active medium is able to recover to its equilibrium state between the consecutive pulse passages.

One more question we have to address here is how the pulse area changes upon reflection from the mirror in our cavity. Suppose that the electric field of the incident pulse is given as:

6 $$\begin{aligned} E_{inc}(t,z) = \frac{1}{2} {\mathscr {E}}_{inc}(t,z) e^{i(\omega _0 t - kz)} + \textit{c.c.}, \end{aligned}$$

with slowly varying pulse envelope ${\mathscr {E}}_{inc}(t,z)$ and central pulse frequency $\omega _0$. Besides that, we assume that the mirror is located at $z=0$ and denote as $\Phi _{inc}$ and $\Phi _{ref}$ the areas of the pulse incident on the mirror and reflected from the mirror respectively. Let multiply both sides of Eq. (6) by $e^{-i \omega _0 t}$ and integrate over time from $-\infty$ to $+\infty$:

7 $$\begin{aligned} \int _{-\infty }^{+\infty } E_{inc}(t',0) e^{-i \omega _0 t'} dt' = \frac{1}{2} \int _{-\infty }^{+\infty } {\mathscr {E}}_{inc}(t',0) dt' + \frac{1}{2} \int _{-\infty }^{+\infty } {\mathscr {E}}_{inc}(t',0) e^{-2i \omega _0 t'} dt'. \ \ \ \ \ \end{aligned}$$

One can see, that first and second integrals in Eq. (7) represent (up to constant factors) the Fourier component of the incident pulse at the frequency $\omega_0$ and $\Phi _{inc}$ respectively. The third integral can be transformed using integration by parts as:

8 $$\begin{aligned} \int _{-\infty }^{+\infty } {\mathscr {E}}_{inc}(t',0) e^{-2i \omega _0 t'} dt' = {\mathscr {E}}_{inc}(t',0) \frac{e^{-2i \omega _0 t'}}{-2 i \omega _0} \Big |_{-\infty }^{+\infty } + \frac{1}{2i} \int _{-\infty }^{+\infty } \frac{1}{\omega _0} \frac{\partial {\mathscr {E}}_{inc}(t',0)}{\partial t'} e^{-2i \omega _0 t'} dt', \ \ \ \ \ \end{aligned}$$

where the first term on the right-hand side turns to zero due to the finite pulse duration. The commonly used slowly varying envelope approximation (SVEA) states, that:

9 $$\begin{aligned} \frac{1}{\omega _0} \Big | \frac{\partial {\mathscr {E}}_{inc}}{\partial t} \Big | \ll {\mathscr {E}}_{inc}. \end{aligned}$$

Therefore validity of SVEA Eq. (9) would allow us to neglect the second term on the right-hand side of Eq. (7) with respect to the first one. Moreover, the presence of the fast-oscillating factor $e^{-2i \omega _0 t'}$ under the integral sign in Eq. (8) can lead to even smaller values of the second term on the right-hand side of Eq. (7), than it could be expected from Eq. (9). Indeed, from Eq. (9) one would estimate the ratio of two terms on the right-hand side of Eq. (7) to be of the order of $\omega _0 \tau _p \gg 1$. At the same time, if we take for example the envelope of a stationary $\pi$-pulse propagating in the amplifying medium with linear losses^6,10^:

$$\begin{aligned} {\mathscr {E}}_{inc}(t',z) = \frac{A}{\cosh \Big ( \frac{t-z/c}{\tau _p} \Big )}, \end{aligned}$$

then we find:

$$\begin{aligned}& \int _{-\infty }^{+\infty } {\mathscr {E}}_{inc}(t^{\prime},0) dt^{\prime} = \frac{\pi }{2} A \tau _p, \\ & \int _{-\infty }^{+\infty } {\mathscr {E}}_{inc}(t^{\prime},0) e^{-2i \omega _0 t^{\prime}} dt^{\prime} = {\frac{\pi }{2}} {\frac{A \tau _p}{\cos h \pi \omega _0 \tau _p}}, \end{aligned}$$

so that their ratio is:

$$\begin{aligned} {\cosh \pi \omega _0 \tau _p} \approx e^{\pi \omega _0 \tau _p}/2 \quad \text {for} \quad \omega _0 \tau _p \gg 1, \end{aligned}$$

what is much larger as compared to just a factor $\omega _0 \tau _p$.

Considering the above, Eq. (7) finally turns into:

$$\begin{aligned} \Phi _{inc}\approx & {} \frac{4 \pi d_{12}}{\hbar } F_{inc}(\omega _0), \end{aligned}$$

with the Fourier transform of the incident pulse $F_{inc}(\omega )$. The exactly same equality is obtained for the area of the reflected pulse. Therefore the areas of $\Phi _{inc}$ and $\Phi _{ref}$ are simply related through the amplitude reflection coefficient of the mirror $r(\omega )$ at the frequency $\omega _0$, assuming that the response of the mirror is broadband enough:

10 $$\begin{aligned} \Phi _{ref} = r(\omega _0) \Phi _{inc}. \end{aligned}$$

It is worthy noting, that the applicability of the relation Eq. (10) reduces to the applicability of SVEA Eq. (9). For long enough pulses with $\omega _0 \tau _p \gg 1$ SVEA is reasonably justified, while for few-cycle pulses it can not be fulfilled anymore.

**Figure 3:**
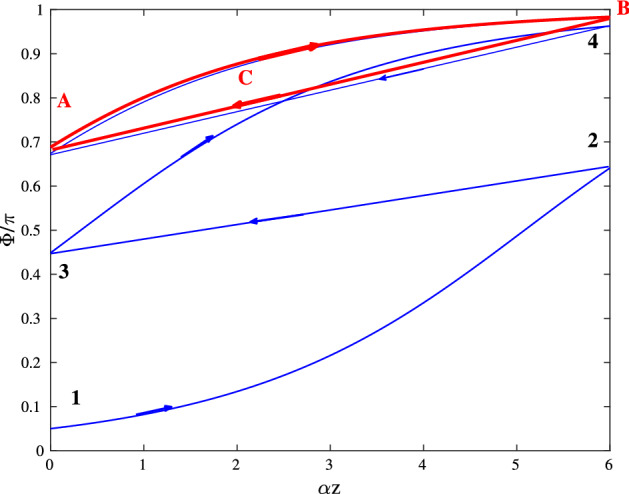
Evolution of the pulse area $\Phi$ in an amplifier medium from the initial value $\Phi _0=0.05 \pi$ to the limit cycle (red curve *ABC*); $r(\omega _0) = 0.7$, $|\alpha |=12$ cm$^{-1}$. This figure was created with Matlab R2016b (http://www.mathworks.com).

Using the area theorem Eq. (2) and branches of it’s solution (similar to that plotted in Fig. 1) we are now able to follow the evolution of the pulse area during a single round-trip in a ring laser cavity. As the pulse propagates in the amplifier, the corresponding point on the diagram is moving from left to right along the amplifier branch from the point 1 to the point 2, see Fig. 3. This propagation is accompanied by the increase of the pulse area. After the pulse passes the amplifier, it is reflected by a non-ideal mirror and its area is thus reduced according to Eq. (10), what corresponds to the moving of the point on the diagram Fig. 3 along the curve 23 from right to left to the point 3. Then, pulse propagates in the amplifier once again along other amplifier branch 34 and so on. One can expect, that after many round-trips a stable self-pulsating regime with pulse having the area in the vicinity of $\pi$, sets up. This limit cycle *ABC* is shown in Fig. 3 with red lines.

This limit cycle can be obtained analytically as following: Let denote as $\Phi _{k}$ the pulse area after *k* round-trips in the cavity, measured at the output of the gain medium in Fig. 2. According to Eqs. (5) and (10), the pulse area after $k+1$ round-trips in the cavity $\Phi _{k+1}$ is related to $\Phi _{k}$ as:

11 $$\begin{aligned} \Phi _{k+1}=2 \arctan \left[ \tan \frac{r \Phi _k}{2} \exp \left( \alpha _0 L_g/2 \right) \right] . \end{aligned}$$

From Eq. (11) one finds the pulse area in the steady-state regime $\Phi ^*$ as:

12 $$\begin{aligned} \Phi ^*=2 \arctan \left[ \tan \frac{r \Phi ^*}{2} \exp \left( \alpha _0 L_g/2 \right) \right] . \end{aligned}$$

If we denote the function on the right-hand side of Eq. (12) as $f(\Phi )$, the stability condition of the steady state $\Phi ^*$ requires:

13 $$\begin{aligned} \Big | \frac{d f}{d \Phi } \Big |_{\Phi =\Phi ^*} < 1. \end{aligned}$$

We note that the stability of the mapping $\Phi ^*$ defined by (13) does not mean automatically the stability of the initial system. It ensures, however, its stability in respect to perturbations with zero frequency.

From Eq. (12) one finds:

14 $$\begin{aligned} \frac{d f}{d \Phi } = \frac{r \exp \left( \alpha _0 L_g/2 \right) }{1 + \sin ^2 \frac{r \Phi ^*}{2} ( \exp \left( \alpha _0 L_g \right) - 1)}. \end{aligned}$$

Therefore the stability condition Eq. (13) yields:

15 $$\begin{aligned} r \exp \left( \alpha _0 L_g/2 \right) < 1 + \sin ^2 \frac{r \Phi ^*}{2} ( \exp \left( \alpha _0 L_g \right) - 1). \end{aligned}$$

If

16 $$\begin{aligned} r \exp \left( \alpha _0 L_g/2 \right) < 1, \end{aligned}$$

Equation (12) has only one non-lasing steady-state solution, $\Phi ^* = 0$ and this solution is stable, since Eq. (13) is satisfied. On the other hand, if

17 $$\begin{aligned} r \exp \left( \alpha _0 L_g/2 \right) > 1, \end{aligned}$$

**Figure 4:**
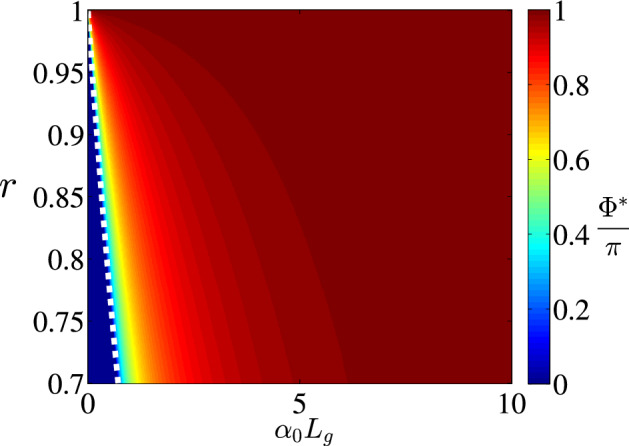
Non-trivial steady-state solution $\Phi ^*$ of Eq. (12) vs. parameters *r* and $\alpha _0 L_g$. White dashed line shows the boundary between domains Eqs. (16) and  (17), i.e. to the left of this boundary non-trivial solution $\Phi ^*$ does not exist. This figure was created with Matlab R2018a (http://www.mathworks.com).

**Figure 5:**
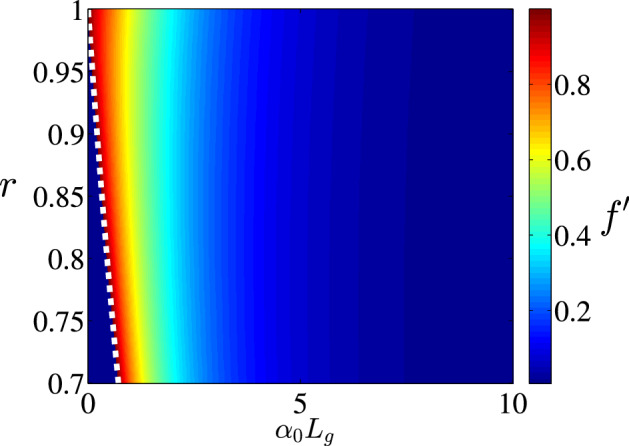
Derivative $f^\prime (\Phi ^*)$ for the non-trivial steady-state solution $\Phi ^*$ of Eq. (12), describing its stability, vs. parameters *r* and $\alpha _0 L_g$. White dashed line shows the boundary between domains Eqs. (16) and  (17), i.e. to the left of this boundary non-trivial solution $\Phi ^*$ does not exist. This figure was created with Matlab R2018a (http://www.mathworks.com).

Equation (12) has two steady-state solutions. The trivial one $\Phi ^* = 0$ is unstable, since Eq. (13) is not fulfilled. Another non-zero solution $0< \Phi ^* < \pi /r$ is shown in Fig. 4 in dependence on the parameters *r* and $\alpha _0 L_g$. Figure 4 shows, that the stationary solution $\Phi ^*$ approaches $\pi$ with increase of *r* or $\alpha _0 L_g$. This steady state is always stable (in the sense of Eq. (14)). Indeed, since the derivative Eq. (14) is larger than 1 for $\Phi = 0$ and smaller than 1 for $\Phi = \pi /r$, at the intermediate point $\Phi ^*$ the derivative Eq. (14) must be smaller than 1, otherwise the equality Eq. (12) could not take place. This fact is demonstrated in Fig. 5, where the derivative Eq. (14) is plotted.

## Numerical simulations

The diagrammatic technique presented above gives a qualitative picture of the evolution of the pulse area in a single-section laser with a unidirectional lasing regime in an inhomogeneously broadened media. For homogeneously broadened media, the area theorem does not hold anymore. Nevertheless, here we show that basically the same dynamics takes place in the homogeneously broadened media as well. Besides, we reveal the details of bifurcation scenario as well as the scaling behavior of the pulse with the pump, and the relation between the pulse durations in a single-and two-section cavities. For this, we will perform direct numerical simulations of the laser equations.

For our simulations, we used the set of Maxwell-Bloch equations describing propagation of light in a two-level amplifying medium under the slowly-varying envelope and rotating-wave approximations^6,8,10,26,27,60^:

18 $$\begin{aligned}&\partial _tp_s(z,t)=-\gamma _2p_s(z,t) + gn(z,t)A(z,t), \end{aligned}$$

19 $$\begin{aligned}&\partial _tn(z,t)=-\gamma _1\left[ n(z,t)-n_0(z)\right] -F(z,t), \end{aligned}$$

20 $$\begin{aligned}&\partial _t A(z,t) + c\partial _zA(z,t)= \kappa p_{s}(z,t), \end{aligned}$$

where $g(z)=\frac{d_{12}(z)}{2\hbar }$, $\kappa =4\pi \omega _0 d_{12} N_0(z)$, $F(z,t) = 4g(z) A(z,t)p_s(z,t)$, $p_s(z,t)$ is the slowly-varying envelope of the imaginary part of the non-diagonal element of the density matrix of a two-level particle, *n*(*z*, *t*) is the population difference between the lower and upper energy levels of a two-level particle, $n_0(z)=-1$ is the stationary value of *n*(*z*, *t*) in the absence of the pulse for amplifier ($n_0=1$ for the absorber), *A*(*z*, *t*) is the real-valued slowly-varying amplitude of the cos-component of the electric field. The parameters of the two-level particles are the transition dipole moment $d_{12}$, concentration of the particles in the gain medium $N_0$, relaxation times $T_1=1/\gamma _1$ and $T_{2}=1/\gamma _2$ as well as the eigen-frequency of the medium $\omega _0$. The set of equations Eqs. (18)–(20) allows accurate modeling of the evolution of extended two-level media in a cavity, assuming relatively long pulse durations and intensities at which the Rabi frequency $\Omega _R \ll \omega _0$, so that no multilevel dynamics come into play. The equations naturally take into account longitudinal multi-mode dynamics and the accompanying nonlinear coherent effects. In Eqs. (18)–(20), we dropped the equations for the real part of the non-diagonal element of the density matrix $p_c(t)$ and the sine-component of the electric field *A*_s_(*z*, *t*)^5^, since in the case of the resonant light-matter interaction $p_c=0$, and hence $A_s=0$^6^.

In the example that will be considered below, the following parameters were used: the wavelength $\lambda = 0.7 \mu m$, the reflection coefficient of the mirror $r = 0.8$, the cavity length $L= 3$ cm, the length of the gain section $L_g= 1$ cm, the transition dipole moment $d_{12} = 5$ Debye, $T_1 = 0.5$ ns, $T_2 = 0.25$ ns.

First, we preformed a set of simulations with gradially increased the concentration of the active particles $N_0$, each time starting simulations from non-lasing state perturbed by noise. We found the first threshold at around $N_0=N_t\approx 6.7\times 10^{10}$ cm$^{-3}$. After this first threshold, the laser was operating in a CW mode. At a value of $N_0=N_f\approx 0.165\cdot 10^{14}$ cm$^{-3}$, small pulsations in the CW mode appeared, indicating its destabilization. This second threshold thus takes place at rather high values of pump $N_f/N_t\approx 250$. This is to be compared to the estimation for RNGH instability threshold, given by^29–31^

21 $$\begin{aligned} N_f/N_t\approx 4\pi ^2c^2 T_1 T_2/L^2\approx 490. \end{aligned}$$

Our numerical result is comparable with this estimation, although somewhat lower. Taking into account that Eq. (21) is only an estimation and was derived for a cavity with distributed parameters, whereas in our case the parameters change significantly across the cavity, we think that the CW instability in the second threshold does correspond to RNGH.

**Figure 6:**
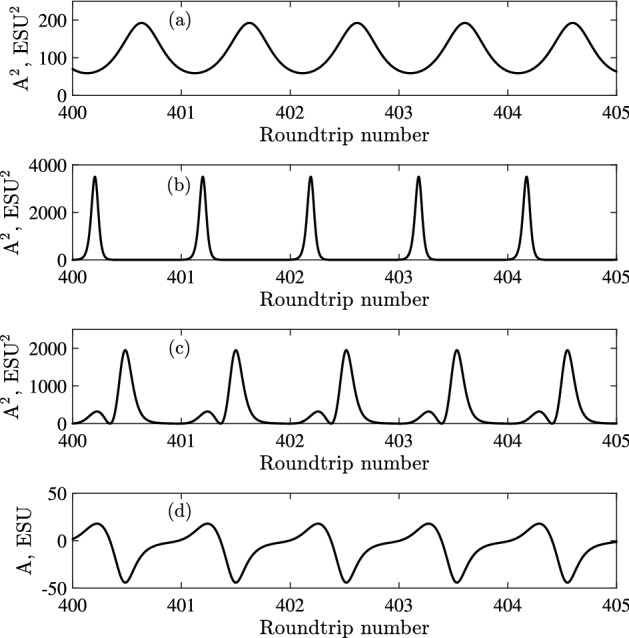
Examples of numerical solutions in regular mode-locking regime. (**a**) Intensity time trace $A^2$ near the second threshold at $N_0 = 0.17\cdot 10^{14}$ cm$^{-3}$; (**b**) A mode-locked regime with a single pulse in the cavity at $N_0 = 0.45\cdot 10^{14}$ cm$^{-3}$; (**c**) and (**d**) dependence of $A^2$ and *A* in the case of two coupled pulses per roundtrip at $N_0 = 0.5\cdot 10^{14}$ cm$^{-3}$. This figure was created with Matlab R2018a (http://www.mathworks.com).

With further increase of the concentration, self-pulsations turn into a pronounced mode-locking regime with a single pulse per roundtrip, see Fig. 6a,b.

**Figure 7:**
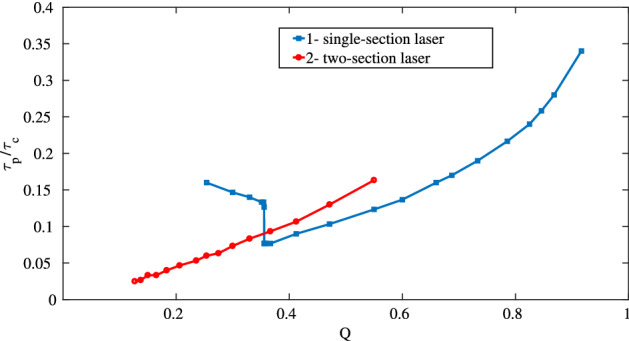
Dependence of the pulse duration $\tau _p$ normalized to the round-trip time $\tau _c$ for a single-section [curve 1 (blue)] and two-section [curve 2 (red)] laser on *Q*, the reciprocal pump excess, normalized to the second threshold of the single-section laser. This figure was created with Matlab R2016b (http://www.mathworks.com).

Above the second threshold, the dependence of the pulse duration on the concentration of amplifying particles $N_0$ was calculated numerically and is shown in Fig. 7. There, the curve 1 shows the dependence of the FWHM pulse duration $\tau _p$ (normalized to the cavity round-trip time $\tau _c=L/c$) on the reciprocal pump $Q=\left( \frac{N_0}{N_f}\right) ^{-1}$ normalized to its value at the second threshold (of a single-section laser) $N_f$. In the region of *Q* from 0.4 to 0.8, the dependence is close to linear, which demonstrates a characteristic feature of CML: the pulse duration decreases with increasing power^24,25^. Up to $Q \approx 0.4$, the lasing takes the form of a single pulse. An example solution is shown in Fig. 6b. At $Q \approx 0.35$, the nature of the solutions changes. In addition to the main pulse, a lower intensity pulse appears. These are two coupled pulses of the $0\pi$-pulse type, that is, the envelope changes its sign. An example of such pulse is given in Fig. 6c,d. With further increase of $N_0$, that is, decrease of *Q*, the solution becomes irregular, but then settles to a harmonic mode-locking with two pulses in the cavity (not shown in the figure). Then, the solution becomes irregular again, after which regular solutions with three pulses in the cavity show up. This scenario with increasing the number of pulses, followed by an irregular regime, repeats itself (not shown).

**Figure 8:**
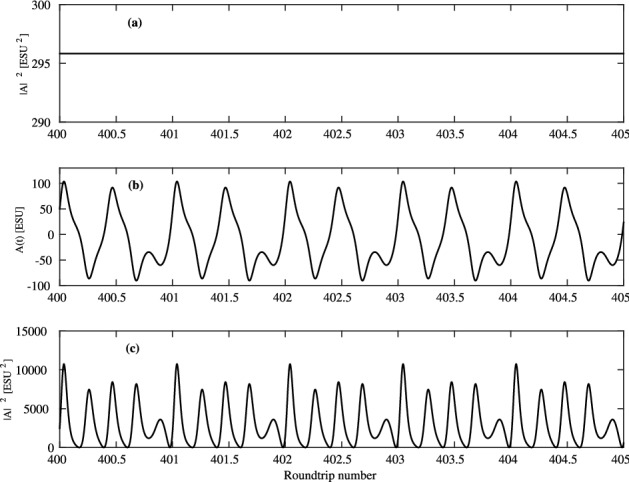
Dynamics for smaller $T_2$, out of the coherent mode-locking regime. (**a**) Intensity-time trace $A^2$ for $N_0$ the same as in Fig. 6b and $T_2=25$ ps (10 times smaller than in Fig. 6b). (**b**) *A*(*t*), and (**c**) $A^2(t)$ for $T_2=25$ ps and $N_0 = 0.45\cdot 10^{15}$ cm$^{-3}$, 10 times larger than in (**a**), providing the same excess above threshold as in Fig. 6b. Other parameters are the same as in Fig. 6. This figure was created with Matlab R2016b (http://www.mathworks.com).

It is interesting to compare the dynamics to the case of smaller $T_2$, that is, out of the coherent regime. Such comparison is made in Fig. 8. In Fig. 8a a simulation is shown with the same parameters as in Fig. 6b, but with 10 times smaller $T_2$. This results in a CW regime, since the excess over the first threshold also decreases, according to Eq. (4). To return back to the same excess over the lasing threshold we need to increase the pump by the same ratio. As it is seen in Fig. 8b,c, this leads to irregular pulsations. From these simulations we see that the mode-locking in the coherent regime (for large $T_2$) is more stable and survives higher pump levels, than the mode-locking in a laser with small $T_2$. In comparison, if we increase $T_1$, keeping all other parameters fixed, then the pulse duration, as given by numerical simulations, increases, since, due to decrease the pump rate $N_0/T_1$, the overall power also decreases.

**Figure 9:**
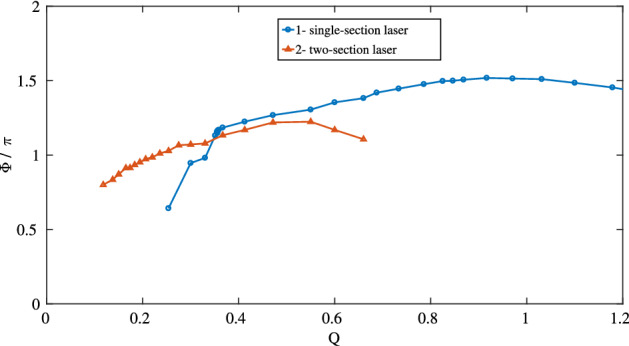
Dependencies of the pulse area at the output of the amplifying medium on Q for a single- (curve 1) and for a two-section laser (curve 2). The area was calculated numerically as the integral of the envelope over the complete roundtrip. This figure was created with Matlab R2016b (http://www.mathworks.com).

Furthermore, we compared our simulations of a single-section laser with a laser, containing both an amplifier and absorber and working in the CML regime. The length of the absorber section was taken to be the half of the length of the amplifier, the concentration was three times less than in the amplifier section, and the dipole moment was twice larger. This twofold difference in the dipole moments is necessary for the implementation of coherent mode-locking in a two-section laser^7^. The relaxation times were taken as: $T_1 = 0.2$ ns, $T_2 = 0.1$ ns. In contrast to a single-section laser, self-pulsations start at the higher pump level and exist in the range from $Q = 0.57$ to $Q = 0.1$ (see Fig. 7, curve 2). After that, the mode-locking regime becomes unstable, and several pulses appear in the generation. With further increase of $N_0$, harmonic mode-locking was observed. As in the case of a single-section laser, the instability zones alternated with harmonic mode-locking zones took place. Comparison of the curves 1 and 2 in Fig. 7 shows that in the given example, the minimum pulse durations in the mode-locking regime differ 3 times between single- and two-section lasers. That is, an absorber allows to reduce the pulse duration, in comparison to the single-section laser without absorber. This happens via more effective preventing the development of “tails” of the pulses that arise in a single-section laser, that is, via better protecting the non-lasing background after the pulse against perturbations. However, the achievable decrease of the pulse durations is not so dramatic, as it could be expected. In both cases, the area of the pulse after the amplifying medium in the mode-locking zone was close to $\pi$. The corresponding dependence is shown in Fig. 9.

The area in Fig. 9 was calculated numerically as the integral over the pulse envelope over the whole roundtrip. Such definition does not allow to determine the area of the pulses when multiple of them are present in the cavity. On the other hand, in this way we can continue the definition of the area to the self-pulsing regimes with a small amplitude and even to the CW regime. With a such defined area, numerical simulations revealed another remarkable feature of a single-section laser: near the second threshold, the pulse area is still close to $\pi$, staying still slightly larger than this value. It even slightly increases with the increase of the pump. After exceeding the second threshold, when self-pulsations start, the area begins to decrease. In the region where the stable mode-locking is achieved, the area is smaller than $\pi$. The dependence of the area on the pump for a two-section laser is also nonmonotoneous: at large durations (low pumps) the area grows but then begins to decrease. Nevertheless, it also stays close to $\pi$.

As we see, our numerical simulations give, in general, pulses with the area around $\pi$, similarly with the result for inhomogeneous broadened media predicted by a mapping in the previous section. Nevertheless, some differences to the mapping-based solutions do exist. First, in contrast to the mapping, the area can exceed the value of $\pi$. Also, differently from the mapping, direct numerical simulations are able to give us the pulse shape which can significantly vary with the pump level. In particular, the mapping predicts, that the solutions with the area around $\pi$ arise directly at the first threshold. On the other hand, it says nothing about the corresponding pulse durations. Our results indicate that those nonzero solutions born at threshold according to the mapping may correspond to the CW solution described in this section.

The mechanism, determining stable self-starting mode-locking in our single-section laser is essentially the same as in the two-section CML laser described in^26^ (because, as mentioned before, the second (absorber) section only introduces some stabilizing effect, without altering the dynamics). Namely, the passage of a $\pi$-pulse leaves nearly all the atoms of the gain section in the ground state. During the roundtrip time, the pump ensures that the population relaxes back. In this situation, if the cavity length is selected properly, the medium has enough time to relax before the next pulse comes. If the cavity length is too long (or, putting it in the other way, the pump is too strong), the number of pulses over the roundtrip time increases as was described before.

## Condition for few-cycle pulse generation in single-section laser

The consideration above suggests that, in order to decrease the pulse duration, we need to decrease the cavity length. In this respect, it is useful to establish a general scaling of Eqs. (18)–(20) which would allow us to rescale existing solutions to the shorter pulse durations. For this, let us suppose that all times in our system are decreased by a factor *K*: $t\rightarrow t/K, T_1\rightarrow T_1/K, T_2\rightarrow T_2/K$, except the transition frequency $\omega _0$ (and thus the wavelength $\lambda$) which we keep the same. This is possible since $\omega _0$ only appears in $\kappa$, so we compensate this by modifying another variable entering $\kappa$ only, as discussed below. The physical meaning of *n* and $p_s$ requires that they remain intact by the rescaling: $n\rightarrow n$, $p_s\rightarrow p_s$. In order to keep balance in Eq. (20), we need to rescale the space coordinate in the same way as time $z\rightarrow z/K$. This means that all intracavity elements, including the whole cavity length, must be also reduced *K* times. Besides, we keep *g* the same. From Eq. (18) we immediately obtain that $A\rightarrow KA$. This, in turn, means, that in Eq. (20) we need to rescale $\kappa$ as $\kappa \rightarrow K^2 \kappa$. We can achieve this by a corresponding change of $N_0$: $N_0\rightarrow K^2 N_0$. This all defines a rescaling, which, being applied to Eqs. (18)–(20), leaves the equations unchanged; also all the possible regimes including mode-locking remain intact. For a mode-locking regime, the pulse duration decreases *K* times, but the pulse shape does not change, and the ratio $\tau _p/\tau _c$ of the pulse duration $\tau _p$ to the cavity length $\tau _c$, as well as the pulse area, remain the same. We note that this is not the only rescaling which is possible in Eqs. (18)–(20), but we find this particular one the most suitable for practical realizations.

**Figure 10:**
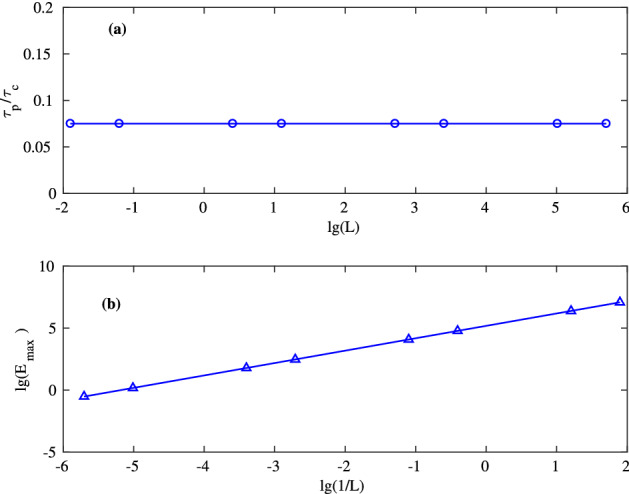
(**a**) Dependence of the pulse duration $\tau _p$ normalized to the cavity round-trip time $\tau _c$ on the cavity length in log scale. (**b**) Dependence of the pulse maximum on the inverse cavity length. This figure was created with Matlab R2016b (http://www.mathworks.com).

We explored this scaling by direct numerical simulations as illustrated in Fig. 10. The dependence in Fig. 10a shows that $\tau _p/\tau _c$ remains constant as we modify the cavity length (together with the other parameters as prescribed by the scaling). Note the logarithmic scale in Fig. 10, allowed to vary *L* from 3 m to 3 mm. Figure 10b shows the dependence of the maximum pulse amplitude on the cavity length as we change *L* according to the rescaling. This curve reveals that  $E_\text {max}$ grows with exactly the same rate as 1/*L* as suggested by the scaling.

Such rescaling makes it possible, using the simulations above, to “rescale” the existing solutions and thus to estimate the parameters of the laser, at which the mode-locking with the pulse duration we want, takes place. By that, we should not however cross the boundary of the validity of the slow envelope approximation (that means that we must consider pulses of at least several cycles in duration), since the rescaling mentioned above does not work anymore for the equations free from the slow envelope. Taking the target pulse duration to be 10 optical cycles (23 fs) and assuming $\tau _p/\tau _c = 0.08$ (cf. Fig. 7), using our rescaling one can obtain $K=34$ in respect to the configuration in Fig. 6b, thus the cavity and the gain section lengths should be about 0.88 mm, and 0.29 mm correspondingly, with $T_1 = 1.4$ ps, $T_2 = 0.7$ ps, and $N_0 = 5.3\cdot 10^{18}$ cm$^{-3}$. We note that the pulse repetition rate in such a short cavity should be as high as 0.34 THz. We checked these parameters by numerical simulations and indeed found a stable mode-locking with required pulse duration, and the dynamics similar to Fig. 6b, and with 34 times higher amplitude.

Although such short pulses are formally supported by Eqs. (18)–(20), the practically achievable pulse duration in every physical realization will be most probably limited by further physical processes. In particular, it is not easy to realize relaxation times in ps range needed for such few-cycle pulses; besides, large pump powers in the range of hundreds Watts will be required in this situation, most probably leading to heating and related problems. Finally, to realize a traveling-wave cavity of 0.1 mm-scale length is also rather challenging.

## Conclusions

To summarize, we have demonstrated that a stable, self-starting coherent mode-locking regime is possible in a single-section laser, containing only an amplifying section. Coherent mode-locking, taking place if the decay time $T_2$ exceeds the pulse duration, was up to now known to appear in lasers containing both absorbing and amplifying sections. Nevertheless, if the cavity length and the pump/loss balance are tuned properly, that is, in such a way that the relaxation after the pulse passage is matched to the fast population change during the pulse, the resulting mode-locking is so stable, that the absorbing section is not needed anymore and can be removed. The self-starting behaviour is ensured since at the required pump levels both non-lasing and CW regimes are highly unstable. On the other hand, as our results show, in the coherent regime (large $T_2$) the pulsations are much more stable than in the incoherent case.

In the article, for inhomogeneously broadened media, we established the existence of the coherent mode-locking and its stability (to zero-frequency perturbations) by constructing a mapping, based on the area theorem Eq. (2). In the case of nonzero frequency detuning between the pulse and the medium, the chirped pulse area theorem should be used^61^. It yields exactly the same equation for the evolution of the pulse area as Eq. (2), just with slightly different definition of the pulse area. Therefore, all results from the “CML in a single-section laser and the area theorem” section of our manuscript should hold for the respective chirped pulse area as well.

For homogeneously broadened media, we have shown the existence and stability of the mode-locking using direct simulations of Maxwell-Bloch equations. In this latter case we established that as the pump increases, mode-locking arises from a CW regime via self-pulsations caused by RNGH instability.

In the mode-locking regime, the pulse area is around $\pi$, that is, a half of the Rabi oscillation. Taking into account that just before the pulse arrival the medium is almost fully inverted, and just after the pulse passage this whole energy is fully transferred into radiation, this regime requires unusually strong pump levels. In the examples considered above, the pump needed for mode-locking exceeds the lasing threshold by hundreds of times. Such high levels might look completely unrealistic for, for instance, semiconductor lasers with electrical pumping, but other media/schemes such as optically pumped gases, or alkali metal vapors, or optically pumped quantum dots, could be promising candidates. This is supported by pulsed regimes already observed in the gas- and vapor based lasers^6,22–25,51–55^. We note that CML is in fact dissipation-less in the sense that all the pump energy can be converted into radiation. So, if the parasitic dissipation channels such as heating and other non-radiative processes are suppressed, the high excess above threshold should not posses a problem. As it was mentioned before, most promising candidates in this respect are gases and vapors. Also, an interesting possibility could be superfluidic helium, since in superfluidic phase the coupling to phonons is suppressed.

Using the scaling established here we showed that, at the cost of reducing the cavity length and increasing the pump power, the pulse durations even in few-cycle range can be obtained. The general scaling obtained by us is the following: to reduce the pulse duration *K* times, the cavity length and roundtrip times $\tau _c$ should be decreased by factor of *K*, accomplished by an increase $K^3$ times of the pump power $N_0/\tau _c$. Besides, relaxation times must be decreased *K* times as well. Since even shorter, single-cycle, pulses were predicted to be achievable with CML in a two-section cavity^11,12^, we expect that this can be also possible for the single-section scheme. This problem requires however rather different theoretical approach and is beyond the scope of the paper.
